# Sleep quality, BDNF genotype and gene expression in individuals with chronic abdominal pain

**DOI:** 10.1186/s12920-014-0061-1

**Published:** 2014-10-31

**Authors:** Swarnalatha Y Reddy, Nat A Rasmussen, Nicolaas H Fourie, Rebecca S Berger, Angela C Martino, Jessica Gill, Ryan Longchamps, Xiao Min Wang, Margaret M Heitkemper, Wendy A Henderson

**Affiliations:** Division of Intramural Research, National Institute of Nursing of Research, National Institutes of Health, Department of Health and Human Resources, 10 Center Drive, Building 10, 2-1341, Bethesda, MD 20892 USA; University of Washington, School of Nursing, Seattle, WA USA

**Keywords:** Sleep, Gene expression, BDNF, Chronic abdominal pain

## Abstract

**Background:**

Sleep quality and genetics may contribute to the etiology of gastrointestinal (GI) symptoms. Individuals with impaired sleep often have a number of associated symptoms including chronic abdominal pain (CAP). The current study examined the interactions of brain-derived neurotrophic factor (BDNF) genotype with sleep quality in persons with CAP and healthy controls. In addition, associations among sleep quality, BDNF genotype, and gene expression were explored in the participants.

**Methods:**

Data were collected on 59 participants (46% male, 61% White, 26.9 ± 6.6 years; CAP (n=19) and healthy controls (n=40)). Participants with CAP reported poorer sleep quality compared to healthy controls. BDNF genotype, categorized as Val/Val homozygotes versus the Met carriers.

**Results:**

Microarray analysis found twenty-four differentially expressed genes by a two-fold magnitude in participants with poor sleep quality compared to good sleep quality, and seven differentially expressed genes comparing CAP to healthy control. Three specific genes in the pain group overlap with sleep quality, including insulin-like growth factor 1 (IGF1), spermatogenesis associated serine-rich 2-like (SPATS2L), and immunoglobulin heavy constant gamma 1 or mu (IGHG1/// IGHM). BDNF was shown to have an interaction effect with GI and sleep symptoms.

**Conclusions:**

Participants with CAP reported poor sleep quality compared to healthy controls. The role of the BDNF Met allele on differential gene expression was not distinct as main factor, but impacted interactions with sleep quality and CAP. Down-regulation of IGF1, SPATS2L, and IGHG1 expression may be related to the etiology of poor sleep quality and CAP.

**Trial registration:**

Clinicaltrial.gov # NCT00824941

**Electronic supplementary material:**

The online version of this article (doi:10.1186/s12920-014-0061-1) contains supplementary material, which is available to authorized users.

## Background

Chronic abdominal pain (CAP) affects an estimated 15 − 20% of people worldwide [1,2], and is a symptom common to functional gastrointestinal (GI) disorders [3-5] such as irritable bowel syndrome (IBS). GI disorders are estimated to cost the United States approximately $30 billion annually related to lost productivity and medical care [6-8]. Sleep is necessary to health and well-being and, too little or too much sleep may increase the risk of developing chronic conditions [9,10]. Sleep disturbances are also associated with increased IBS symptoms [11-14]. In subjects with IBS, sleep scores are significantly correlated with pain [14].

There is evidence that genetics predispose people to alterations in sleep architecture and pain [9,10,15,16]. Brain-derived neurotrophic factor (BDNF) is encoded by the BDNF gene, which contains a single nucleotide polymorphism (SNP) Val66Met (rs6265), where adenine and guanine alleles vary, resulting in a variation of valine to methionine at codon 66. Studies by Bachmann et al. [16] show that BDNF Val66Met polymorphism modulates sleep intensity in healthy humans. As measured by electroencephalogram the study found pronounced variations in slow wave sleep activity specific to BDNF. Another study indicated that elevated BDNF expression in IBS patients compared to healthy controls is correlated with higher abdominal pain scores [17]. Although there are limited studies involving IBS and BDNF, a 10-biomarker index that included BDNF, was used to differentiate IBS from non-IBS subjects and found to be sensitive and specific [18].

The purpose of this study was twofold: (a) to compare sleep quality between participants with CAP and healthy controls and (b) to explore interactions among sleep quality, BDNF alleles, and gene expression in participants with CAP and healthy controls. It was expected that findings would provide new insights into the role of gene expression in relation to sleep quality and chronic pain conditions.

## Methods

### Design and setting

The study was approved by the Institutional Review Board and the Office of Human Subjects Research at the NIH. Participants were exclusively recruited to a natural history protocol (09-NR-0064, Clinicaltrial.gov # NCT00824941) conducted at the Hatfield Clinical Research Center, NIH. Details on participant’s recruitment, inclusion and exclusion criteria are provided in Additional file 1. Participants gave written consent and questionnaires were collected for sleep measurements during outpatient visits from January 2009 to July 2011. The peripheral blood samples were collected from fasting participants during the same visit.

### Sample demographics and clinical variables

Demographics and clinical characteristics, and the sleep measures of the participants are given in Tables 1 and 2. Healthy controls met the inclusion criteria for age, menses (females) and had no history of abdominal pain. Also, healthy controls had no other organic disorders or GI diseases (e.g., inflammatory bowel disease, celiac disease, biliary disorders, bowel resection) and had no cardiac, pulmonary, neurologic, renal, endocrine, or gynecological pathology and had no other exclusions (see Additional file 1). CAP is defined as a self-reported history of greater than 6 months of abdominal pain of unknown origin and no known diagnosed organic cause for the abdominal pain. This information is determined by self-report and confirmed by medical record review.

**Table 1:** **Demographics and clinical characteristics of the healthy controls and participants with chronic abdominal pain (CAP)**

**Variable**	**Total**	**CAP**	**Healthy control**	**p-value**
	**(n= 59)**	**(n= 19)**	**(n= 40)**	
**Gender (n)**				-
Females	32 (54%)	14 (74%)	18 (45%)	
Males	27 (46%)	5 (26%)	22 (55%)	
**Race (n)**				-
Black	10 (17%)	3 (16%)	7 (17.5%)	
Asian	10 (17%)	2 (11%)	8 (20%)	
White	36 (61%)	13 (68%)	23 (57.5%)	
Mixed	3 (5%)	1 (5%)	2 (5%)	
**Age**	26.93 ± 6.64	26.68 ± 4.77	27.05 ± 7.42	0.821
Range (yrs)	(13–45)	(14–33)	(13–45)	
**Body Mass Index (BMI)**	25.34 ± 5.26	25.31 ± 4.49	25.35 ± 5.64	0.974
Range (kg/m^2^)	(18.65 - 43.22)	(20.19 - 35.07)	(18.65 - 43.22)	

Age and BMI values are reported as mean ± standard deviation.

**Table 2:** **Sleep quality measures of healthy controls and participants with chronic abdominal pain (CAP)**

**PSQI measures**	**Total**	**CAP**	**Healthy control**	**p-value**
	**(n=59)**	**(n=19)**	**(n= 40)**	
**Global PSQI score**	4.63 ± 0.41	6.42 ± 0.80	3.78 ± 0.40	0.006*
**Sleep latency**	0.98 ± 0.12	1.42 ± 0.21	0.78 ± 0.14	0.014*
**Sleep disturbances**	1.10 ± 0.07	1.32 ± 0.13	1.00 ± 0.07	0.046*
**Daytime dysfunction**	0.69 ± 0.10	1.16 ± 0.21	0.48 ± 0.10	0.006*
**Medications to sleep**	0.19 ± 0.08	0.47 ± 0.22	0.05 ± 0.05	0.077
**Overall sleep quality**	0.92 ± 0.09	1.11 ± 0.15	0.83 ± 0.10	0.131
**Duration of sleep**	0.39 ± 0.09	0.47 ± 0.18	0.35 ± 0.11	0.558
**Sleep efficiency**	0.36 ± 0.11	0.47 ± 0.23	0.30 ± 0.11	0.507

PSQI: Pittsburgh Sleep Quality Index.

Data are reported as mean ± standard deviation; *Significant findings (p-value ≤0.05).

Gender, race, and age (in years) were collected using the Socio-demographic Questionnaire developed by the Center for Research in Chronic Disorders, University of Pittsburgh School of Nursing (1999) and administered through the Clinical Trials Database. During the clinical visit, the participant’s weight was measured in triplicate and height was measured in duplicate. The average height and weight were used to determine body mass index (BMI), calculated as weight in kilograms divided by height in meters squared (Table 1).

### Sleep measurement

Participants rated their sleep quality by using the PSQI [19] questionnaire, which provides a subjective measure of sleep quality over the past month. The questionnaire contains 19 self-reported questions and 5 questions completed by a bed partner. The latter 5 questions are used for clinical information only, not tabulated in the scoring of the PSQI. The 19 self-reported questions are grouped into seven component scores: 1) sleep latency, 2) sleep disturbances, 3) daytime dysfunction, 4) medications to sleep, 5) overall sleep quality, 6) duration of sleep, and 7) sleep efficiency. The seven component scores were summed to obtain a global PSQI score, ranging from 0–21 (Table 2). Component scores range from 0 (good sleep quality) to 3 (poor sleep quality). Global PSQI score ≤5 is associated with good sleep quality and >5 with poor sleep quality [19]. Buysse and colleagues reported internal consistency reliability for the PSQI for overall component and individual item scores (Cronbach’s α=0.83). Global PSQI scores >5 were reported to have a sensitivity of 89.6% and specificity of 86.5% in comparing poor sleepers to good sleepers [19].

### DNA extraction and BDNF genotyping

Peripheral blood was collected from the participants using a yellow top BD Vacutainer® (BD Biosciences, Franklin Lakes, NJ) and frozen immediately at −80°C until the time of extraction. DNA extraction was performed on 5 mL of blood using the QIAamp DNA Blood Maxi Kit (Qiagen, Frederick, MD) according to the manufacturer’s instructions. If less than 5 mL of blood was available, phosphate buffered saline was added to bring the total volume to 5 mL. DNA concentration was determined by NanoDrop™ 1000 spectrophotometer (Thermo Scientific, Wilmington, DE) and, extracted DNA was stored at −20°C prior to genotyping assay.

Sample DNA was dried overnight on a MicroAmp™ optical 384-Well reaction plate (in duplicates) at a concentration 5 ng/μL. Per genotyping protocol of the manufacturer, Assay ID: C_11592758_10 (product # 4351379), DNA was subsequently combined with the TaqMan® Universal PCR Master Mix and TaqMan® SNP Genotyping Assay Mix (Applied Biosystems, Foster City, CA). The genotyping assay contains two unlabeled primers (forward and reverse) for amplifying the sequence of interest and two labeled probes for detecting alleles. The probes consist of target-specific oligonucleotides with a reporter dye at the 5′- end of each probe, VIC® dye (linked to the 5′-end of the allele 1 probe), 6FAM™ dye (linked to the 5′-end of the allele 2 probe). Also, the probes incorporate nonfluorescent quencher on 3′-end attached to a minor groove binder.

Allelic discrimination PCR reactions (40 cycles) were then performed for BDNF rs6265 on 7900 HT Fast Real-Time PCR System (Applied Biosystems, Foster City, CA). Subsequently, an endpoint fluorescence measurement was obtained with the Sequence Detection Systems (SDS) software of 7900 HT Fast system (Applied Biosystems, Foster City, CA), to examine the samples for discrimination between the specific alleles.

### RNA isolation and amplification

The blood samples (2.5 mL) from each participant were collected in PAXgene (Qiagen, Valencia, CA) tubes and stored at −81°C. Total RNA was extracted and purified from each blood sample using a RNA PAXgene kit (Qiagen, Frederick, MD) according to the manufacture’s protocol and stored at −81°C. The RNA quality and quantity were determined by spectrometry and by using the RNA 6000 Nano LabChip kit on a 2100 Bioanalyzer (Agilent Technologies, Santa Clara, CA). Total RNA that passed quality control criteria was used for microarray and qPCR experiments.

### Microarray processing

Microarrays were processed by one person at the Laboratory of Molecular Technology, National Cancer Institute (NIH, Frederick, MD), following standard operating protocol to minimize non-biologic technical bias. The RNA quantity, purity, and integrity were assessed by a NanoDrop™ 1000 spectrophotometer (Wilmington, DE) and Experion electrophoresis station (Bio-Rad Laboratories, Hercules, CA), respectively, or by 2100 Bioanalyzer using the RNA 6000 Nano LabChip kit (Agilent Technologies, Santa Clara, CA). All samples had a high quality score (RIN >9).

Double-stranded cDNA, was synthesized from 10 ng total RNA followed by a linear isothermal amplification to produce single-strand cDNA, which was direct labeled by biotin using Ovation Whole Blood Solution kit (NuGEN technologies, Inc., San Carlos, CA) following manufacturer’s protocol. Biotinylated cDNA (4.4 μg) from each sample was mixed with the control buffer and hybridized on each Human Genome U133 plus 2.0 array (Affymetrix, Santa Clara, CA) at 45°C for 16 hours in a GeneChip hybridization oven at 60 rpm. GeneChip arrays were washed on Fluidics Station 450 using manufacturer’s recommended scripts and scanned on GeneChip Scanner 3000 (Affymetrix, Santa Clara, CA). GeneChip Command Console (AGCC 3.0, Affymetrix) was used to scan the images and for data acquisition.

To enable the comparison between arrays, a global scaling factor (target signal to 500) was used across all arrays to minimize the variables caused by sample preparation, hybridization/ staining or from different lots of arrays. Microarray quality control was evaluated for each array by examination of background, noise, average signal, % present, ratio of signal values for probe sets representing the 5′ and 3′ ends of actin and glyceraldehyde-3-phosphate dehydrogenase transcripts, and total signal for probesets for BioB, BioC, BioD and CreX.

### qPCR experiments

Total RNA (200 ng) was reverse transcribed using the RT^2^ First Strand cDNA Synthesis Kit (Qiagen, Frederick, MD). RT^2^ PCR Primer Assays and RT^2^ SYBR Green PCR Master Mix (Qiagen, Frederick, MD) were used to analyze trends in the gene expression of IGF-1, IGHG1, and SPATS2L. These genes were significantly expressed comparing CAP to healthy control, and also comparing poor quality sleep to good sleep quality. All reactions were plated in triplicate and beta actin (known as ACTB) was used as a housekeeping gene. The PCR plate was stored overnight wrapped in tin foil at 4°C and then run on 7900 HT real-time cycler (Applied Biosystems, Foster City, CA).

### Data analysis

Descriptive statistics for all demographic, clinical variables and sleep quality measures were calculated using SPSS Statistics (IBM SPSS Inc., Chicago, IL) (Tables 1 and 2). Comparisons were made for age and BMI between the two levels of pain group (CAP versus healthy control) of the participants using t-tests. For sleep quality measures, a priori p-values <0.05 were considered significant (Table 2).

For the analysis of genotyping data, the threshold for Quality value, which influences the allele call was set to 100 (Additional file 2: Table S1). Chi-square tests were performed to study the associations of BDNF SNP with categorical measures of sleep quality (poor sleep versus good sleep) and pain groups (CAP versus healthy control). The BDNF Met allele has previously been associated and implicated in various behavioral and cognitive processes [20-22]. In order to assess the associations of the Met allele with the sleep, pain and gene expression data, heterozygous and homozygous Met genotypes were grouped together into a Met carrier group (Table 3) for comparison with the homozygous Val genotype (or non-Met carrier group) as is typically done in the study of these alleles due to the low frequency of the homozygous Met genotype [20,22].

**Table 3:** **BDNF genotyping (N= 49 participants) and the clinical measures of pain and sleep quality**

**ID**	**BDNF**	**Pain**	**Sleep quality**
3	Val/Val	Healthy Control	Poor quality
6	Val/Val	Healthy Control	Good quality
7	Val/Val	Healthy Control	Good quality
11	Val/Val	CAP	Good quality
12	Val/Val	Healthy Control	Poor quality
14	Val/Val	Healthy Control	Good quality
16	Val/Val	CAP	Poor quality
17	Val/Val	Healthy Control	Good quality
18	Val/Val	Healthy Control	Poor quality
20	Val/Val	Healthy Control	Poor quality
22	Val/Val	Healthy Control	Good quality
25	Val/Val	Healthy Control	Good quality
26	Val/Val	Healthy Control	Good quality
28	Val/Val	Healthy Control	Poor quality
31	Val/Val	Healthy Control	Good quality
35	Val/Val	Healthy Control	Good quality
36	Val/Val	Healthy Control	Good quality
38	Val/Val	CAP	Poor quality
39	Val/Val	Healthy Control	Good quality
40	Val/Val	Healthy Control	Good quality
41	Val/Val	CAP	Poor quality
42	Val/Val	CAP	Good quality
44	Val/Val	CAP	Good quality
46	Val/Val	CAP	Good quality
49	Val/Val	Healthy Control	Good quality
51	Val/Val	Healthy Control	Good quality
52	Val/Val	Healthy Control	Good quality
55	Val/Val	CAP	Good quality
57	Val/Val	Healthy Control	Good quality
61	Val/Val	CAP	Poor quality
64	Val/Val	CAP	Poor quality
65	Val/Val	CAP	Good quality
66	Val/Val	CAP	Good quality
69	Val/Val	CAP	Poor quality
1	Met Carrier	Healthy Control	Good quality
2	Met Carrier	Healthy Control	Poor quality
8	Met Carrier	Healthy Control	Good quality
15	Met Carrier	Healthy Control	Good quality
24	Met Carrier	Healthy Control	Good quality
27	Met Carrier	CAP	Good quality
29	Met Carrier	Healthy Control	Good quality
32	Met Carrier	CAP	Poor quality
33	Met Carrier	Healthy Control	Good quality
34	Met Carrier	Healthy Control	Poor quality
37	Met Carrier	Healthy Control	Good quality
43	Met Carrier	CAP	Poor quality
45	Met Carrier	CAP	Poor quality
53	Met Carrier	CAP	Poor quality
56	Met Carrier	Healthy Control	Good quality

Microarray data on 26 participants (a subset of 59 sample full cohort, Table 4 and Additional file 2: Table S2) were analyzed for gene expression patterns using Partek Genomics Suite software (Partek Inc., St. Louis, MO). The probe-level robust multichip average background correction, quantile normalization and Log_2_ transformation followed by probe-set summarization were performed on gene expression intensity values. Multi-way ANOVA was used to compare the gene expression profiles between the two levels of, ***Pain***: *CAP* (11) and *healthy control* (15), ***Sleep quality***: *poor sleep quality* (7) and *good sleep quality* (19) and ***BDNF***: *Val homozygous (*17) and *Met carriers* (9). ***Gender*** (Females 15 and Males 11) was included in the analysis. The interaction terms of sleep quality * pain, sleep quality * BDNF, and pain * BDNF were included in the 4-way ANOVA model. Differentially expressed gene lists were generated for sleep quality and pain groups by a magnitude of 2-fold change (in either direction), and at the false discovery rate (FDR) 0.05 level. The same fold change criteria was used to obtain differentially expressed genes for BDNF groups, but with unadjusted p-values <0.05 as FDR criteria were not fulfilled.

**Table 4:** **Sample cohort (N= 26) for microarray experiments comprising BDNF genotype and the clinical measures of pain and sleep quality**

**ID**	**BDNF**	**Pain**	**Sleep quality**
3	Val/Val	Healthy Control	Poor quality
6	Val/Val	Healthy Control	Good quality
11	Val/Val	CAP	Good quality
16	Val/Val	CAP	Poor quality
22	Val/Val	Healthy Control	Good quality
25	Val/Val	Healthy Control	Good quality
35	Val/Val	Healthy Control	Good quality
36	Val/Val	Healthy Control	Good quality
38	Val/Val	CAP	Poor quality
39	Val/Val	Healthy Control	Good quality
40	Val/Val	Healthy Control	Good quality
41	Val/Val	CAP	Poor quality
44	Val/Val	CAP	Good quality
46	Val/Val	CAP	Good quality
49	Val/Val	Healthy Control	Good quality
52	Val/Val	Healthy Control	Good quality
55	Val/Val	CAP	Good quality
8	Met Carrier	Healthy Control	Good quality
24	Met Carrier	Healthy Control	Good quality
27	Met Carrier	CAP	Good quality
29	Met Carrier	Healthy Control	Good quality
32	Met Carrier	CAP	Poor quality
37	Met Carrier	Healthy Control	Good quality
43	Met Carrier	CAP	Poor quality
45	Met Carrier	CAP	Poor quality
56	Met Carrier	Healthy Control	Good quality

Interactive pathway analysis (Qiagen Systems, Redwood City, CA) was performed on differentially expressed genes between poor sleep quality and good sleep quality to identify gene networks associated with specific biological functions. Genes were colored based on expression values, up-regulated in red and down-regulated in green, respectively. Connectivity of differentially expressed genes was made via the molecules added from Ingenuity knowledge database. A network was generated based on significant differentially expressed genes of CAP compared to healthy control.

The qPCR data on genes IGF-1, IGHG1, and SPATS2L were uploaded and analyzed with the use of Web Analysis tool (http://www.sabiosciences.com/). Threshold cycle cut-off was set at 35 cycles per literature recommendation and trends in gene expression were analyzed in respect to a 95% confidence interval.

## Results and discussion

The sample comprised 59 participants (46% male, 61% White, 26.9 ± 6.6 years), which included individuals with CAP (n=19) and healthy controls (n=40) (Table 1). A comparison of age and body mass index (BMI) showed no significant differences between CAP and healthy controls (Table 1). Sleep quality measures [19] of the participants are given in Table 2. As expected, participants with CAP had significantly higher scores than healthy controls on the global Pittsburgh Sleep Quality Index (PSQI), sleep latency, sleep disturbances, and daytime dysfunction scores. Higher sleep scores on the PSQI scale equate to poorer sleep quality. Also, there was a trend toward higher scores for medications to sleep and overall sleep quality in participants with CAP compared to healthy controls (Table 2). However, there were no significant differences in measures of duration of sleep or sleep efficiency between the two groups. These results are consistent with studies reported in literature on relationships between sleep quality and IBS [14,23,24].

Genotyping studies were employed to explore BDNF SNP (rs6265) on the sleep quality (poor sleep versus good sleep) and pain (CAP versus healthy control) groups. The raw data from genotyping experiment processed on 59 samples is given in Additional file 2: Table S1. The Quality value reflects the probability of the genotype call or Val66Met polymorphism. Data were dichotomized as the homozygous Val group (i.e., Val/Val; GG alleles) and Met carriers consisting of genotypes supporting the Met allele (i.e., Val/Met (GA alleles) and Met/Met (AA alleles). Depending on the detection of SNPs, data on 49 samples were used for analysis, given in Table 3. There were no significant associations observed with regard to BDNF SNP with sleep quality or pain grouping.

Details of the cases and healthy controls of the sample cohort used for microarray experiments are given in Table 4. Also, the demographics of the microarray sample cohort are provided in Additional file 2: Table S2. There were no significant differences between the microarray cohort and the full cohort with regard to gender, age, or BMI. The distribution of BDNF genotypes are known to vary among races [25-28]. Our study included a substantial Caucasian cohort which was selected as a statistically viable group for an analysis of both SNP and gene expression data while controlling for the possible effects of race.

**Figure 1:**
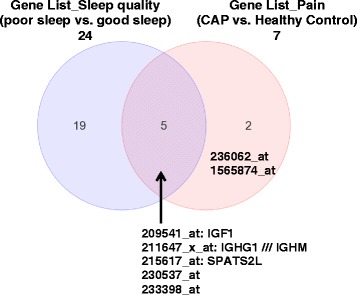
**Intersection of two lists of significant genes.** Venn diagram showing the overlap of most differentially expressed genes of the sleep quality (poor sleep vs. good sleep, total of 24) and pain groups (CAP vs. healthy control, total of 7). The gene names and probeset IDs are provided for the pain group, and also that overlap with the sleep quality group. Although 5 genes overlap, gene names are available for three: IGF1, SPATS2L, and IGHG1///IGHM.

Gene expression studies by microarray showed majority of differentially expressed genes comparing poor sleep quality to good sleep quality group were down-regulated (by a two-fold magnitude, Table 5). Interestingly, for the pain group, the CAP had increased gene expression (>2.0 fold) compared to healthy controls (Table 6). Three specific named genes overlap in the sleep quality (poor sleep versus good sleep) and pain (CAP versus healthy control) groups: insulin-like growth factor 1 (IGF1), spermatogenesis associated, serine-rich 2-like (SPATS2L), and immunoglobulin heavy constant gamma 1 or mu (IGHG1//IGHM). Also, two unnamed genes (probesets 230537_at and 233398_at) overlap between sleep quality and pain groups (Figure 1).

**Table 5:** **Microarray differentially expressed genes for the sleep quality group that passed FDR (5%) and fold change (>2.0 and< −2.0) criteria (subset of 26 participants)**

**Probeset ID**	**Gene symbol**	**Gene title**	**p-value sleep quality (poor sleep vs. good sleep)**	**Fold-change sleep quality (poor sleep vs. good sleep)**
232011_s_at	MAP1LC3A	microtubule-associated protein 1 light chain 3 alpha	2.39E-05	2.3261
1555019_at	CDHR1	cadherin-related family member 1	2.59E-05	2.1365
230537_at	---	---	4.27E-05	−2.0029
209541_at	IGF1	insulin-like growth factor 1 (somatomedin C)	8.64E-06	−2.0056
223595_at	TMEM133	transmembrane protein 133	4.85E-07	−2.0090
229116_at	CNKSR2	connector enhancer of kinase suppressor of Ras 2	4.74E-05	−2.0228
203830_at	C17orf75	chromosome 17 open reading frame 75	2.21E-05	−2.0231
223275_at	PRMT6	protein arginine methyltransferase 6	4.54E-05	−2.0384
218875_s_at	FBXO5	F-box protein 5	2.39E-05	−2.1082
225153_at	GFM1	G elongation factor, mitochondrial 1	1.06E-05	−2.1148
1556096_s_at	UNC13C	unc-13 homolog C (C. elegans)	3.98E-05	−2.1205
219443_at	TASP1	taspase, threonine aspartase, 1	1.50E-05	−2.1483
211647_x_at	IGHG1 /// IGHM	immunoglobulin heavy constant gamma 1 (G1m marker) /// immunoglobulin heavy constant mu	7.63E-07	−2.2431
1552972_at	LOC100507431	uncharacterized LOC100507431	9.61E-06	−2.2919
215617_at	SPATS2L	spermatogenesis associated, serine-rich 2-like	1.23E-05	−2.3091
225114_at	AGPS	alkylglycerone phosphate synthase	2.78E-05	−2.3288
213929_at	EXPH5	exophilin 5	3.60E-06	−2.3403
206557_at	ZNF702P	zinc finger protein 702, pseudogene	3.18E-05	−2.5112
201689_s_at	TPD52	tumor protein D52	2.21E-05	−2.5938
211635_x_at	IGHA1 /// IGHA2 /// IGHD /// IGHG1 /// IGHG3 /// IGHG4 /// IGHM /// IGHV4-31	immunoglobulin heavy constant alpha 1 /// immunoglobulin heavy constant alpha 2 (A2m ma	4.26E-05	−2.6440
209891_at	SPC25	SPC25, NDC80 kinetochore complex component, homolog (S. cerevisiae)	6.20E-05	−2.8255
211634_x_at	IGHM	immunoglobulin heavy constant mu	4.61E-05	−3.1417
214973_x_at	IGHD	immunoglobulin heavy constant delta	6.75E-05	−4.6680

FDR: False Discovery Rate.

**Table 6:** **Microarray differentially expressed genes for the pain group that passed FDR (5%) and fold change (>2.0 and< −2.0) criteria (subset of 26 participants)**

**Probeset ID**	**Gene symbol**	**Gene title**	**p-value pain (CAP vs. healthy control)**	**Fold-change pain (CAP vs. healthy control)**
*215617_at	SPATS2L	spermatogenesis associated, serine-rich 2-like	4.28E-06	2.5283
*230537_at	---	---	3.41E-06	2.3876
*233398_at	---	---	3.95E-06	2.3134
*209541_at	IGF1	insulin-like growth factor 1 (somatomedin C)	2.55E-06	2.1813
236062_at	---	---	2.52E-06	2.1507
*211647_x_at	IGHG1 /// IGHM /// LOC100133862	immunoglobulin heavy constant gamma 1 (G1m marker) /// immunoglobulin heavy constant mu	2.81E-06	2.1102
1565874_at	---	---	2.22E-06	2.02869

FDR: False Discovery Rate.

*Probesets (genes) of pain group overlap with Sleep quality group.

**Figure 2:**
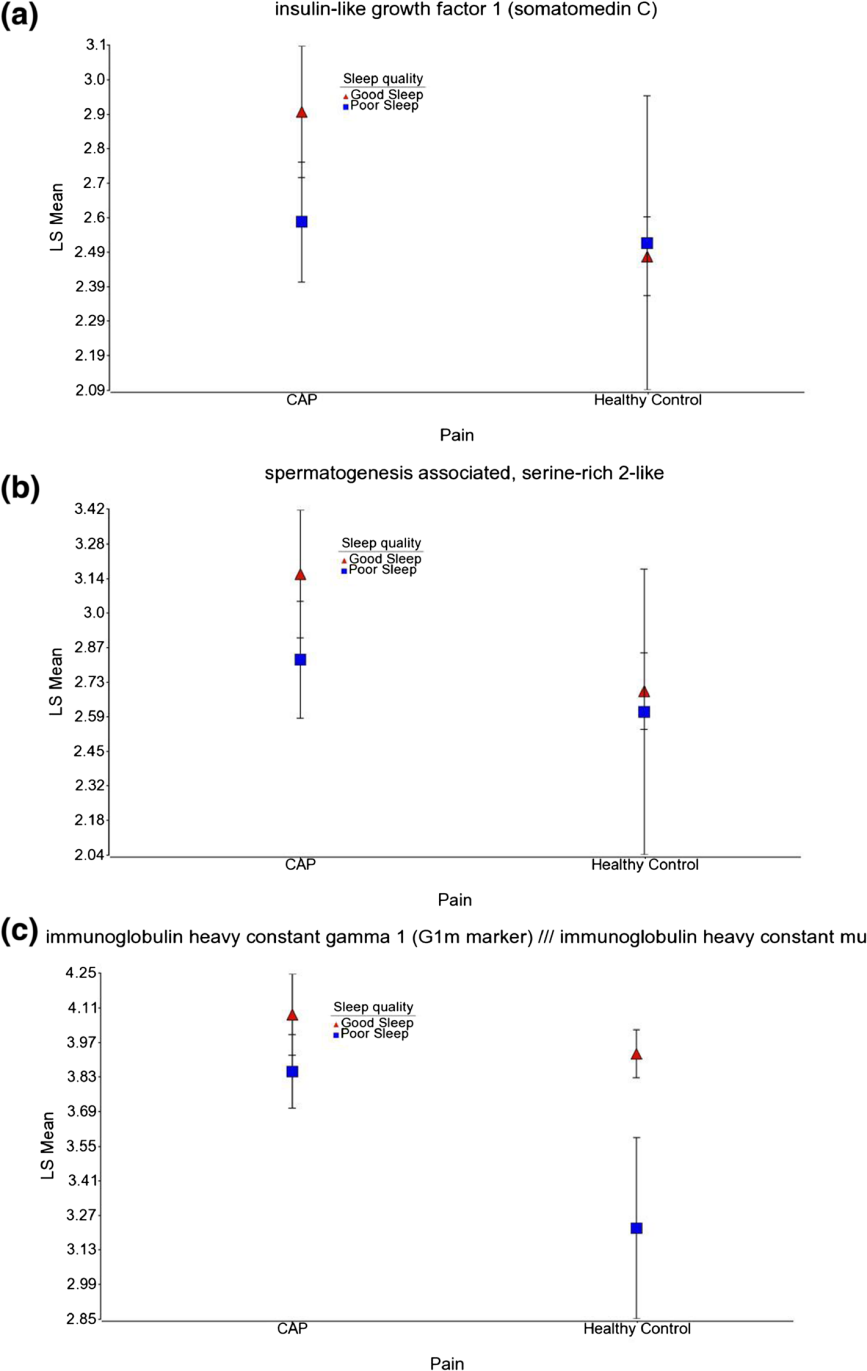
**Expression patterns of IGF1, SPATS2L, and IGHG1 /// IGHM for sleep and pain groups.** Distribution of the intensity values for sleep quality and pain groups of the differentially expressed gene: **(a)** IGF1, **(b)** SPATS2L and, **(c)** IGHG1 /// IGHM. The y-axis represents the log_2_ normalized least square mean of the gene expression intensity. The samples are colored by sleep quality (good sleep in red and poor sleep in blue).

The intensity of specific genes such as IGF1, SPATS2L, and IGHG1 /// IGHM are shown across the two groups of pain and sleep quality (Figure 2). An examination of gene expression profiles associated with CAP showed decreased values for poor sleep quality compared to good sleep quality. In healthy controls, the difference in gene expression was minimal based on sleep quality for IGF1 and SPATS2L genes (Figures 2a and 2b).

**Figure 3:**
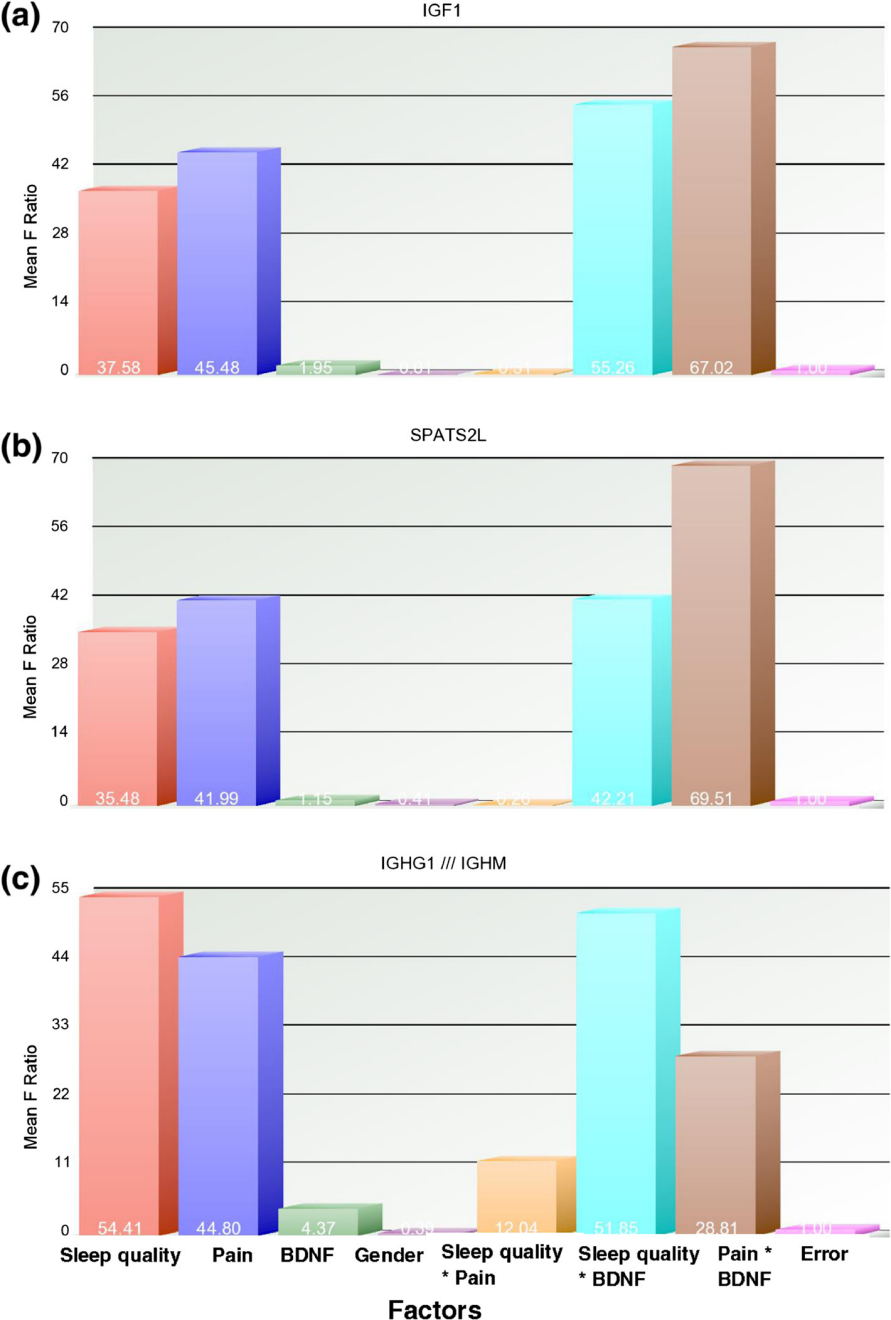
**Sources of variation of the significant genes.** Effect of various factors and the interactions across the differentially expressed genes: **(a)** IGF1, **(b)** SPATS2L, and **(c)** IGHG1 /// IGHM. Factors of ANOVA model and random error are on the x-axis and mean squared F Ratio (measure of variance) of the gene on the y-axis. Average mean square values for each factor and interaction are provided.

Differentially expressed genes for BDNF group, comparing Val homozygous to Met carriers are given in Additional file 2: Table S3. The most prominent down-regulated genes are human leukocyte antigen, major histocompatibility complex, class II, DQ alpha 1(HLA-DQA1) and urotension 2 (UTS2). HLA-DQA1 plays an important role in immune system and is expressed in antigen presenting cells. Variations of HLA-DQA1 gene have been associated with an increased risk of celiac disease. UTS2, a neuropeptide expressed only in brain tissue, is implicated in regulation of sleep stages [29]. Differentially expressed genes of BDNF groups are found to have no overlap with differentially expressed genes of either pain group or sleep quality group. An examination of the main factors (BDNF, sleep quality, and pain) and their interactions on differential gene expression of IGF1, SPATS2L, and IGHG1/// IGHM (Figure 3) indicates that BDNF has an indirect effect, which is additive to the model.

Real time PCR was used to validate the microarray results. Participants with CAP had up-regulation in the expression of IGF1, IGHG1 and SPATS2L (2.83-fold, 1.36-fold and 1.05-fold respectively) compared to healthy controls. The three genes showed fold change by a factor of 0.252, 0.735, and 0.950 for IGF1, IGHG1 and SPATS2L, respectively (95% CI: 0.00001, 2.64; 0.00001, 5.18; 0.00001, 6.42).

**Figure 4:**
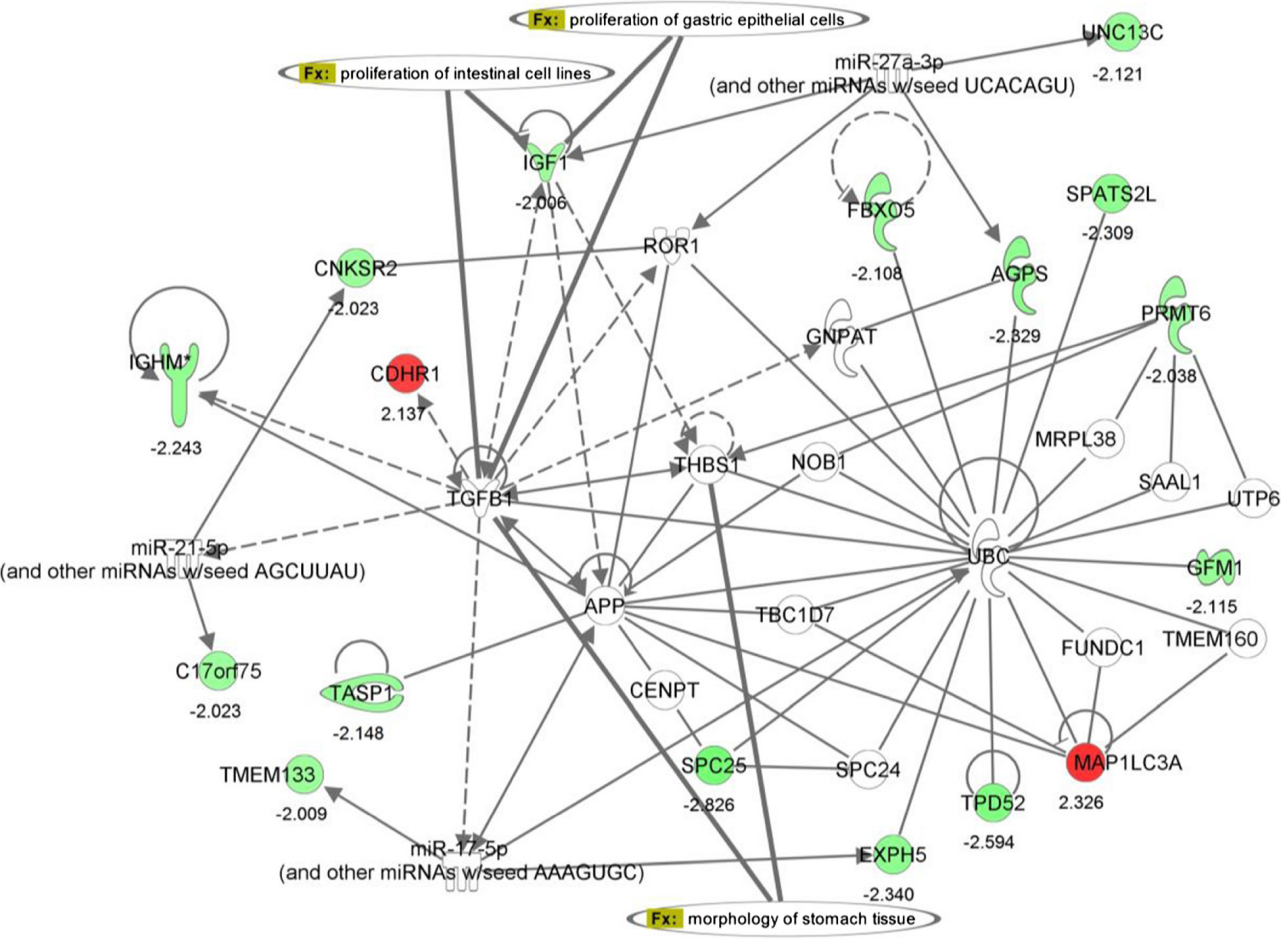
**Functional network associated with the differentially expressed genes.** Schematic diagram of a gene network related to differential expression of sleep quality group (good sleep versus poor sleep). Coloring of genes is based on differential expression (fold changes are shown), down-regulation in green and, up-regulation in red. Genes with no coloring are added from Ingenuity knowledge database. Direct and indirect relationships are shown by solid and dashed lines, respectively. The arrow indicates specific directionality of interactions. Gene associations with some of the digestive system development functions are shown.

Interactive pathway analysis (IPA) (Qiagen Ingenuity Systems, Inc., Redwood City, CA) on differential expressed genes comparing poor sleep quality to good sleep quality resulted in a network associated with functions such as cellular development, hematopoiesis, and tissue morphology. A schematic diagram of functional gene network is shown in Figure 4, of which some have a role in gastrointestinal diseases. Differentially expressed genes are colored (up-regulated in red and down-regulated in green). The genes uncolored are absent in the differentially expressed gene list, but have relationships as shown in the network. The growth factor IGF1 appears pivotal, involved with the proliferation of intestinal cell lines and gastric epithelial cells (Figure 4).

**Figure 5:**
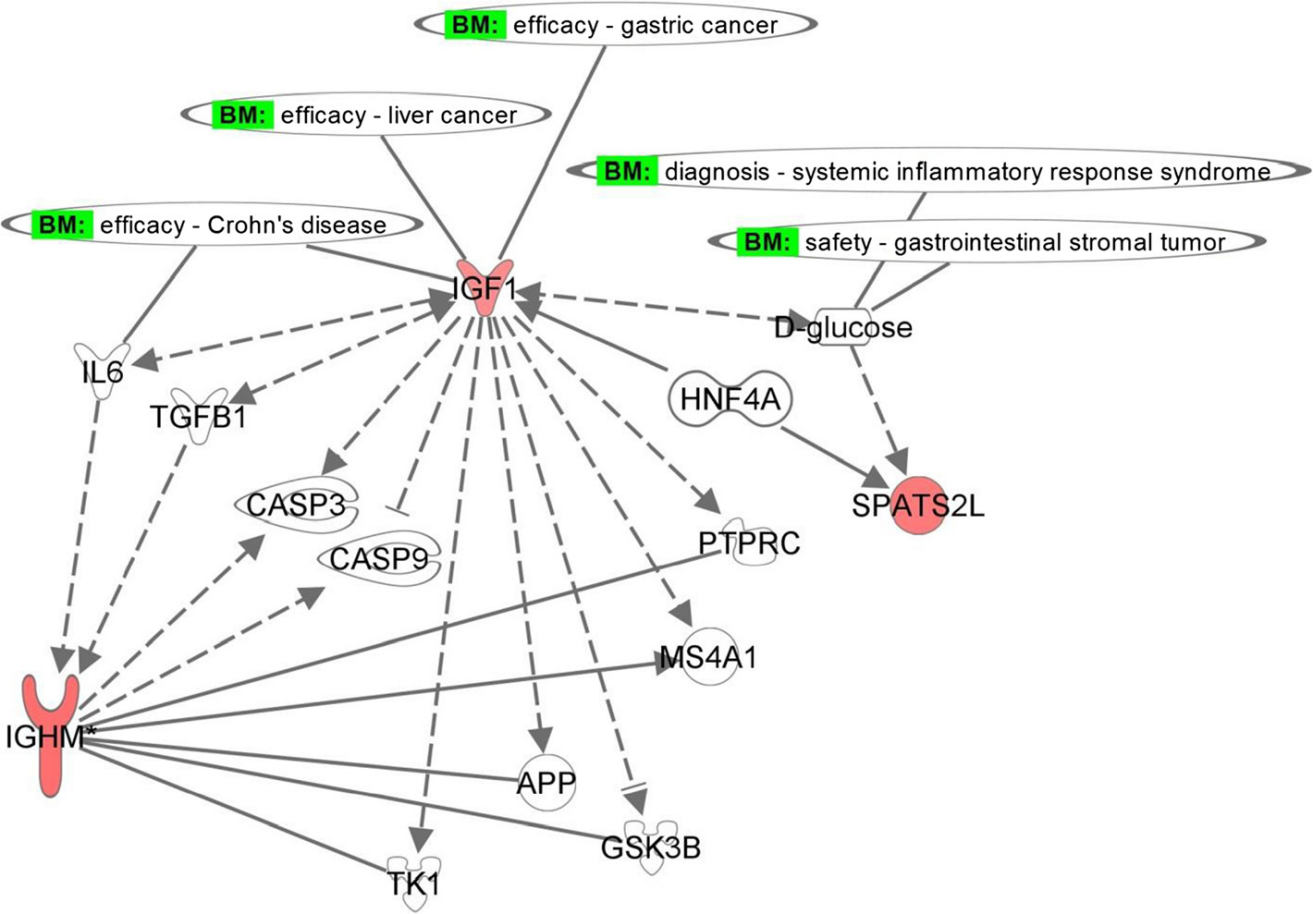
**Molecular paths and interactions of the significant genes.** Specific interactions for the genes IGF1, IGHM, and SPATS2L were generated from Ingenuity knowledge database related to gastrointestinal diseases. Red coloring indicates up-regulated genes for the pain group (CAP vs. healthy control). Direct and indirect relationships are shown by solid and dashed lines, respectively. Potential biomarkers are shown.

Molecular paths generated based on the most significant genes of pain group, IGF1, SPATS2L, and IGHM are shown in Figure 5. SPATS2L is a gene of unknown function, involved in protein-protein interactions, binding to Ubiquitin (UBC) protein (Figures 4), and regulated by D-glucose [30]. SPATS2L, along with IGF1, have a direct interaction with a nuclear receptor, hepatocyte nuclear factor 4 alpha (HNF4A) and an indirect interaction with D-glucose (Figure 5). Mutant LOC26010 (SPATS2L) gene with SNP substitution, allelic variation: A/G (rs2881745) was shown to be associated with arthritis in humans [31]. Another significant gene, IGHM is a trans-membrane receptor and has an important role in B cell development and signaling [32]. IGHM has a direct interaction with amyloid-β precursor protein (APP), the latter in indirect interaction with IGF1 (Figures 4 and 5).

Of the 3 genes of interest, more has been published on the relationship between IGF1 and sleep [33-35]. Growth hormone is important, because reductions in this neuropeptide are associated with increased sleep latency, as well as increases in wakening, and reduced duration of sleep [34]. Decreased levels of serum IGF1 are associated with excessive daytime sleepiness [33], and sleep apnea [34,35] in patients compared to healthy controls.

A growing number of studies show subclinical inflammation in IBS, therefore potentially relating to similar findings of a reduction of serum IGF1 in inflammatory bowel diseases [36,37]. The results of our study are relevant in that IGF1 is an important initiator of cellular repair processes, induces intestinal and linear growth, and may be linked to chronic inflammatory gastrointestinal pathway [38,39]. Also, IGF1 was shown to have a protective effect against inflammation in the bowel [40] and found to be therapeutic in patients with inflammatory bowel disease [40-42].

## Conclusions

Participants with CAP reported poor sleep quality compared to healthy controls. The role of the BDNF Met allele as main effect on gene expression is not obvious although sleep and pain have an influence on differential gene expression. However, the interactions of Met allele with sleep quality or pain impacts differential gene expression. Poor sleep quality in those with CAP may be related to the down-regulation of IGF1, SPATS2L, and IGHG1. A larger sample size with balanced study designs in future studies is needed to better delineate the relationships between BDNF and sleep quality or BDNF and pain. Future research targeting the associations of sleep quality in participants with co-morbid GI conditions is imperative.

## **Additional files**

Additional file 1:
**Inclusion and Exclusion Criteria of Study Participants in Clinical Protocol.**
Additional file 2: Table S1. Raw data on genotyping of BDNF (rs6265) processed on 384-Well reaction plate for 59 participants. **Table S2.** Demographics and clinical characteristics of the sample cohort used for microarray experiments. **Table S3.** Microarray differentially expressed genes for the BDNF group with fold change criteria (>2.0 and < -2.0) and unadjusted p-values (< 0.05) for a subset of 26 participants.

## Abbreviations

ACTB: β − ACTin
APP: Amyloid-β Precursor Protein
BDNF: Brain-Derived Neurotrophic Factor
cDNA: Complementary DNA
CAP: Chronic Abdominal Pain
FDR: False Discovery Rate
GI: Gastrointestinal
HNF4A: Hepatocyte Nuclear Factor 4 Alpha
IBS: Irritable Bowel Syndrome
IGF1: Insulin-like Growth Factor 1
IGHG1: Immunoglobulin Heavy Constant Gamma 1 or Mu
IL-6: Interleukin 6
IPA: Interactive Pathway Analysis
mL: Milliliter
μL: Microliter
μg: Microgram
ng: Nanogram
PSQI: Pittsburgh Sleep Quality Index
qPCR: Quantitative real-time polymerase chain reaction
rhIGF1: Recombinant Human Insulin-like Growth Factor 1
rpm: Revolutions Per Minute
SNP: Single Nucleotide Polymorphism
SPATS2L: Spermatogenesis Associated, Serine-rich 2-like
SPSS: Statistical Product and Service Solution
