# Supplementary material for: Sleep quality, BDNF genotype and gene expression in individuals with chronic abdominal pain
Source: BMC Med Genomics. 2014 Oct 31;7:61. doi: 10.1186/s12920-014-0061-1 (PMC4226913; doi:10.1186/s12920-014-0061-1)
Supplement: Additional file 1: — Inclusion and Exclusion Criteria of Study Participants in Clinical Protocol. [file 12920_2014_61_MOESM1_ESM.doc]

Additional file 1: Inclusion and Exclusion Criteria of Study Participants in Clinical Protocol

| Ages Eligible for Study: | 13 Years to 45 Years |
| --- | --- |
| Genders Eligible for Study: | Both |
| Accepts Healthy Volunteers: | Yes |

**INCLUSION CRITERIA**:

To be included, patients must have met all of the following:

- Have a history of abdominal pain for greater than 6 months
- Males ages 13-45 years old or females ages 13-45 years old who have had their menses for at least 2 years
- Provide written informed consent, prior to entering the study or undergoing any study procedures

**EXCLUSION CRITERIA**:

Patients with any of the following were excluded:

- Have a history of an organic GI disease (e.g., inflammatory bowel disease, celiac disease, biliary disorders, bowel resection) cardiac, pulmonary, neurologic, renal, endocrine, or gynecological pathology
- Are currently taking medications for GI symptoms daily such as 5-HT3 antagonists/5-HT4 agonists, prokinetic drugs, laxatives (but not fiber supplements), anti-diarrheals or antispasmodics
- Are currently taking other medications daily that would alter serotonin (e.g., serotonin specific reuptake inhibitors [SSRI]), catecholamines (e.g., tricycle antidepressants but not inhaled beta-agonist for mild-moderate asthma), cortisol (excluding inhaled corticosteroids)
- Work during the late evening and night (as cortisol levels may be altered)
- Severe co-morbid pain or psychiatric conditions (e.g., fibromyalgia, bipolar or psychotic disorder)
- Take greater than 300 mg of caffeine containing beverages or food (e.g. chocolate) in the afternoon-evening or greater than 2 servings of alcohol containing beverages everyday (decaffeinated coffee is acceptable)
- Are unable to give informed consent
- Are unable to physically use the touch screen for the purpose of the study
- Are visually impaired or currently institutionalized
- Females who are pregnant or lactating
