# Supplementary material for: Sleep quality, BDNF genotype and gene expression in individuals with chronic abdominal pain
Source: BMC Med Genomics. 2014 Oct 31;7:61. doi: 10.1186/s12920-014-0061-1 (PMC4226913; doi:10.1186/s12920-014-0061-1)
Supplement: Additional file 2: Table S1. — Raw data on genotyping of BDNF (rs6265) processed on 384-Well reaction plate for 59 participants. Table S2. Demographics and clinical characteristics of the sample cohort used for microarray experiments. Table S3. Microarray differentially expressed genes for the BDNF group with fold change criteria (>2.0 and < -2.0) and unadjusted p-values (< 0.05) for a subset of 26 participants. [file 12920_2014_61_MOESM2_ESM.doc]

**Additional file 2**

**Table S1** Raw data on genotyping of BDNF (rs6265) processed on 384-Well reaction plate for 59 participants

| **Well** | **Sample Name** | **Marker Name** | **Allele X Rn** | **Allele Y Rn** | **Genotype Call*** | **Quality Value** | **Passive Ref** |
| --- | --- | --- | --- | --- | --- | --- | --- |
| 2 | A2 | FAM1 VIC2 | 2.2254748 | 2.9310322 | Both | 100 | 2246.7708 |
| 4 | A4 | FAM1 VIC2 | 2.361473 | 3.04913 | Both | 100 | 2234.8914 |
| 6 | A6 | FAM1 VIC2 | 2.9578943 | 1.5421759 | a2 | 100 | 2471.583 |
| 8 | A8 | FAM1 VIC2 | 0.578384 | 4.3631606 | a1 | 100 | 1987.7273 |
| 10 | A10 | FAM1 VIC2 | 2.9576702 | 1.5325136 | a2 | 100 | 2496.1753 |
| 12 | A12 | FAM1 VIC2 | 2.90769 | 1.5308622 | a2 | 100 | 2423.8113 |
| 14 | A14 | FAM1 VIC2 | 2.966481 | 1.5747074 | a2 | 100 | 2522.4932 |
| 16 | A16 | FAM1 VIC2 | 2.281368 | 3.0729396 | Both | 100 | 2349.4348 |
| 18 | A18 | FAM1 VIC2 | 3.0310502 | 1.5791979 | a2 | 100 | 2440.8472 |
| 20 | A20 | FAM1 VIC2 | 3.0029492 | 1.5283191 | a2 | 100 | 2262.2603 |
| 22 | A22 | FAM1 VIC2 | 3.0040784 | 1.5043396 | a2 | 100 | 2218.6638 |
| 24 | A24 | FAM1 VIC2 | 1.2282102 | 1.3417461 | Undetermined |  | 1998.0942 |
| 26 | B2 | FAM1 VIC2 | 2.2053254 | 2.9468172 | Both | 100 | 2707.3347 |
| 28 | B4 | FAM1 VIC2 | 2.2516873 | 3.0398357 | Both | 100 | 2593.364 |
| 30 | B6 | FAM1 VIC2 | 2.8774123 | 1.5735185 | a2 | 100 | 2848.5493 |
| 32 | B8 | FAM1 VIC2 | 0.5212219 | 4.287732 | a1 | 100 | 2338.6426 |
| 34 | B10 | FAM1 VIC2 | 2.9736245 | 1.5361118 | a2 | 100 | 2723.274 |
| 36 | B12 | FAM1 VIC2 | 2.8772595 | 1.5382329 | a2 | 100 | 2616.6997 |
| 38 | B14 | FAM1 VIC2 | 2.9667075 | 1.5540594 | a2 | 100 | 2522.1543 |
| 40 | B16 | FAM1 VIC2 | 2.2575004 | 2.9776301 | Both | 100 | 2477.7114 |
| **Well** | **Sample Name** | **Marker Name** | **Allele X Rn** | **Allele Y Rn** | **Genotype Call*** | **Quality Value** | **Passive Ref** |
| 42 | B18 | FAM1 VIC2 | 2.9519079 | 1.5330892 | a2 | 100 | 2645.9236 |
| 44 | B20 | FAM1 VIC2 | 2.925282 | 1.5437418 | a2 | 100 | 2689.1575 |
| 46 | B22 | FAM1 VIC2 | 2.907801 | 1.5623978 | a2 | 100 | 2640.858 |
| 48 | B24 | FAM1 VIC2 | 2.8002627 | 1.4572412 | a2 | 100 | 2056.9714 |
| 50 | C2 | FAM1 VIC2 | 2.9904692 | 1.5136666 | a2 | 100 | 2213.2505 |
| 52 | C4 | FAM1 VIC2 | 2.348731 | 2.9834805 | Both | 100 | 1989.5092 |
| 54 | C6 | FAM1 VIC2 | 3.3271177 | 1.4765809 | a2 | 100 | 2000.4465 |
| 56 | C8 | FAM1 VIC2 | 2.9510167 | 1.5475012 | a2 | 100 | 2122.0154 |
| 58 | C10 | FAM1 VIC2 | 2.9346097 | 1.5065918 | a2 | 100 | 2115.1372 |
| 60 | C12 | FAM1 VIC2 | 2.9509804 | 1.497521 | a2 | 100 | 2055.6514 |
| 62 | C14 | FAM1 VIC2 | 3.0864756 | 1.5511336 | a2 | 100 | 2019.7758 |
| 64 | C16 | FAM1 VIC2 | 3.1557677 | 1.5627937 | a2 | 100 | 1929.791 |
| 66 | C18 | FAM1 VIC2 | 2.4605944 | 2.9386678 | Both | 100 | 1872.2241 |
| 68 | C20 | FAM1 VIC2 | 0.6362428 | 4.274501 | a1 | 100 | 1529.1472 |
| 70 | C22 | FAM1 VIC2 | 3.0242596 | 1.5136185 | a2 | 100 | 2184.67 |
| 72 | C24 | FAM1 VIC2 | 3.0980036 | 1.4508641 | a2 | 100 | 1653.7297 |
| 74 | D2 | FAM1 VIC2 | 3.0245173 | 1.540416 | a2 | 100 | 2497.401 |
| 76 | D4 | FAM1 VIC2 | 2.4347012 | 3.0216904 | Both | 100 | 2055.705 |
| 78 | D6 | FAM1 VIC2 | 3.198875 | 1.5552571 | a2 | 100 | 2144.2712 |
| 80 | D8 | FAM1 VIC2 | 3.153346 | 1.545096 | a2 | 100 | 2180.1453 |
| 82 | D10 | FAM1 VIC2 | 3.0467207 | 1.5671777 | a2 | 100 | 2166.29 |
| 84 | D12 | FAM1 VIC2 | 3.10208 | 1.570847 | a2 | 100 | 2095.8367 |
| 86 | D14 | FAM1 VIC2 | 3.206538 | 1.564477 | a2 | 100 | 2065.146 |
| 88 | D16 | FAM1 VIC2 | 3.1801035 | 1.5306863 | a2 | 100 | 2055.211 |
| **Well** | **Sample Name** | **Marker Name** | **Allele X Rn** | **Allele Y Rn** | **Genotype Call*** | **Quality Value** | **Passive Ref** |
| 90 | D18 | FAM1 VIC2 | 2.4201567 | 3.0027962 | Both | 100 | 1953.4441 |
| 92 | D20 | FAM1 VIC2 | 0.7067901 | 4.2188787 | a1 | 100 | 1521.477 |
| 94 | D22 | FAM1 VIC2 | 3.1878824 | 1.5221885 | a2 | 100 | 2088.453 |
| 96 | D24 | FAM1 VIC2 | 3.225889 | 1.4469105 | a2 | 100 | 1492.4067 |
| 98 | E2 | FAM1 VIC2 | 2.39144 | 3.07222 | Both | 100 | 2011.698 |
| 100 | E4 | FAM1 VIC2 | 3.3596168 | 1.4959826 | a2 | 100 | 1947.69 |
| 102 | E6 | FAM1 VIC2 | 2.5903997 | 3.1510537 | Both | 100 | 1897.1472 |
| 104 | E8 | FAM1 VIC2 | 3.1681807 | 1.5293745 | a2 | 100 | 2043.0721 |
| 106 | E10 | FAM1 VIC2 | 3.116262 | 1.5119674 | a2 | 100 | 2073.043 |
| 108 | E12 | FAM1 VIC2 | 2.3940763 | 2.9801304 | Both | 100 | 1969.6711 |
| 110 | E14 | FAM1 VIC2 | 2.3729491 | 3.0593112 | Both | 100 | 1828.9391 |
| 112 | E16 | FAM1 VIC2 | 2.459817 | 3.2034461 | Both | 100 | 1844.3013 |
| 114 | E18 | FAM1 VIC2 | 3.0602882 | 1.5356061 | a2 | 100 | 2015.7804 |
| 116 | E20 | FAM1 VIC2 | 3.301782 | 1.5456502 | a2 | 100 | 1872.3811 |
| 118 | E22 | FAM1 VIC2 | 2.504114 | 3.089739 | Both | 100 | 1844.2815 |
| 120 | E24 | FAM1 VIC2 | 2.908741 | 1.5211476 | a2 | 100 | 1510.594 |
| 122 | F2 | FAM1 VIC2 | 2.268035 | 3.026545 | Both | 100 | 2416.807 |
| 124 | F4 | FAM1 VIC2 | 2.7783322 | 1.5976604 | a2 | 100 | 2498.068 |
| 126 | F6 | FAM1 VIC2 | 2.3247879 | 3.0987177 | Both | 100 | 2311.1584 |
| 128 | F8 | FAM1 VIC2 | 2.9218178 | 1.5665437 | a2 | 100 | 2439.7417 |
| 130 | F10 | FAM1 VIC2 | 3.0122228 | 1.5649935 | a2 | 100 | 2265.4688 |
| 132 | F12 | FAM1 VIC2 | 2.3227732 | 2.9273078 | Both | 100 | 2129.7944 |
| 134 | F14 | FAM1 VIC2 | 2.3727052 | 3.041204 | Both | 100 | 2145.7715 |
| 136 | F16 | FAM1 VIC2 | 2.3569696 | 3.074414 | Both | 100 | 2159.481 |
| **Well** | **Sample Name** | **Marker Name** | **Allele X Rn** | **Allele Y Rn** | **Genotype Call*** | **Quality Value** | **Passive Ref** |
| 138 | F18 | FAM1 VIC2 | 3.057955 | 1.5069022 | a2 | 100 | 2280.2354 |
| 140 | F20 | FAM1 VIC2 | 3.0024767 | 1.5568967 | a2 | 100 | 2150.3882 |
| 142 | F22 | FAM1 VIC2 | 2.4620955 | 3.0090947 | Both | 100 | 2100.092 |
| 144 | F24 | FAM1 VIC2 | 3.1478949 | 1.4894257 | a2 | 100 | 1901.3411 |
| 146 | G2 | FAM1 VIC2 | 2.9940393 | 1.5277888 | a2 | 100 | 2334.2002 |
| 148 | G4 | FAM1 VIC2 | 3.189642 | 1.4964818 | a2 | 100 | 2195.025 |
| 150 | G6 | FAM1 VIC2 | 3.1456263 | 1.5589883 | a2 | 100 | 2134.227 |
| 152 | G8 | FAM1 VIC2 | 3.1271272 | 1.5680103 | a2 | 100 | 1858.7505 |
| 154 | G10 | FAM1 VIC2 | 2.4094148 | 2.929046 | Both | 100 | 2000.2012 |
| 156 | G12 | FAM1 VIC2 | 3.0953376 | 1.5059897 | a2 | 100 | 2070.5898 |
| 158 | G14 | FAM1 VIC2 | 2.331262 | 2.986218 | Both | 100 | 2009.7295 |
| 160 | G16 | FAM1 VIC2 | 3.0849109 | 1.5853238 | a2 | 100 | 2129.516 |
| 162 | G18 | FAM1 VIC2 | 3.2063713 | 1.5760188 | a2 | 100 | 1992.6432 |
| 164 | G20 | FAM1 VIC2 | 3.1251996 | 1.5650604 | a2 | 100 | 2009.0142 |
| 166 | G22 | FAM1 VIC2 | 3.1724591 | 1.5183234 | a2 | 100 | 2082.331 |
| 168 | G24 | FAM1 VIC2 | 3.208937 | 1.5159632 | a2 | 100 | 1714.0698 |
| 170 | H2 | FAM1 VIC2 | 3.1183338 | 1.5243758 | a2 | 100 | 2645.805 |
| 172 | H4 | FAM1 VIC2 | 3.1862772 | 1.5679721 | a2 | 100 | 2439.2244 |
| 174 | H6 | FAM1 VIC2 | 3.1070232 | 1.5957626 | a2 | 100 | 2494.8936 |
| 176 | H8 | FAM1 VIC2 | 3.1296782 | 1.5857913 | a2 | 100 | 2593.2947 |
| 178 | H10 | FAM1 VIC2 | 2.4276743 | 3.0909698 | Both | 100 | 2312.1892 |
| 180 | H12 | FAM1 VIC2 | 3.1147707 | 1.546513 | a2 | 100 | 2303.355 |
| 182 | H14 | FAM1 VIC2 | 2.45774 | 3.152651 | Both | 100 | 2255.609 |
| 184 | H16 | FAM1 VIC2 | 3.2202811 | 1.5315105 | a2 | 100 | 2358.7769 |
| **Well** | **Sample Name** | **Marker Name** | **Allele X Rn** | **Allele Y Rn** | **Genotype Call*** | **Quality Value** | **Passive Ref** |
| 186 | H18 | FAM1 VIC2 | 3.0664654 | 1.5363203 | a2 | 100 | 2512.4778 |
| 188 | H20 | FAM1 VIC2 | 3.2079794 | 1.5464978 | a2 | 100 | 2363.3423 |
| 190 | H22 | FAM1 VIC2 | 3.244203 | 1.5179449 | a2 | 100 | 2276.9048 |
| 192 | H24 | FAM1 VIC2 | 3.010108 | 1.4996842 | a2 | 100 | 2051.1804 |
| 194 | I2 | FAM1 VIC2 | 2.9632237 | 1.5025129 | a2 | 100 | 2541.1863 |
| 196 | I4 | FAM1 VIC2 | 3.0831885 | 1.5268701 | a2 | 100 | 2422.748 |
| 198 | I6 | FAM1 VIC2 | 2.4917939 | 3.1171165 | Both | 100 | 2260.7979 |
| 200 | I8 | FAM1 VIC2 | 3.1209261 | 1.5555047 | a2 | 100 | 2324.4692 |
| 202 | I10 | FAM1 VIC2 | 2.2851732 | 3.1028657 | Both | 100 | 2115.7646 |
| 204 | I12 | FAM1 VIC2 | 3.056499 | 1.5216343 | a2 | 100 | 2175.0308 |
| 206 | I14 | FAM1 VIC2 | 2.3267186 | 3.057616 | Both | 100 | 2046.5437 |
| 208 | I16 | FAM1 VIC2 | 3.0828927 | 1.5515342 | a2 | 100 | 2334.135 |
| 210 | I18 | FAM1 VIC2 | 3.170641 | 1.5430332 | a2 | 100 | 2303.4263 |
| 212 | I20 | FAM1 VIC2 | 3.202662 | 1.5216613 | a2 | 100 | 2313.8423 |
| 214 | I22 | FAM1 VIC2 | 3.149104 | 1.5160941 | a2 | 100 | 2263.6057 |
| 216 | I24 | FAM1 VIC2 | 3.0424442 | 1.5088781 | a2 | 100 | 2067.4255 |
| 218 | J2 | FAM1 VIC2 | 3.084005 | 1.5485535 | a2 | 100 | 2037.031 |
| 220 | J4 | FAM1 VIC2 | 3.0458453 | 1.5326512 | a2 | 100 | 2138.0918 |
| 222 | J6 | FAM1 VIC2 | 2.477288 | 3.020817 | Both | 100 | 2036.6052 |
| 224 | J8 | FAM1 VIC2 | 3.1622846 | 1.5305623 | a2 | 100 | 2031.6417 |
| 226 | J10 | FAM1 VIC2 | 2.4655585 | 3.0897167 | Both | 100 | 1921.4668 |
| 228 | J12 | FAM1 VIC2 | 3.0768032 | 1.5616066 | a2 | 100 | 1912.4556 |
| 230 | J14 | FAM1 VIC2 | 2.5140293 | 3.0533745 | Both | 100 | 1897.2598 |
| 232 | J16 | FAM1 VIC2 | 3.1589468 | 1.4883096 | a2 | 100 | 2034.1772 |
| **Well** | **Sample Name** | **Marker Name** | **Allele X Rn** | **Allele Y Rn** | **Genotype Call*** | **Quality Value** | **Passive Ref** |
| 234 | J18 | FAM1 VIC2 | 3.1477344 | 1.5138209 | a2 | 100 | 2143.111 |
| 236 | J20 | FAM1 VIC2 | 3.0520186 | 1.5510056 | a2 | 100 | 1999.5616 |
| 238 | J22 | FAM1 VIC2 | 3.0332572 | 1.5006714 | a2 | 100 | 2068.5332 |
| 240 | J24 | FAM1 VIC2 | 3.1221774 | 1.5431389 | a2 | 100 | 1683.5908 |
| 242 | K2 | FAM1 VIC2 | 3.1133976 | 1.5164769 | a2 | 100 | 1940.4902 |
| 244 | K4 | FAM1 VIC2 | 3.0917518 | 1.5550565 | a2 | 100 | 1917.3644 |
| 246 | K6 | FAM1 VIC2 | 3.3374505 | 1.5482165 | a2 | 100 | 1650.4421 |
| 248 | K8 | FAM1 VIC2 | 2.5248783 | 3.1649065 | Both | 100 | 1643.145 |
| 250 | K10 | FAM1 VIC2 | 3.1241982 | 1.5335428 | a2 | 100 | 1912.759 |
| 266 | L2 | FAM1 VIC2 | 2.8733392 | 1.5866227 | a2 | 100 | 2148.6057 |
| 268 | L4 | FAM1 VIC2 | 3.1029086 | 1.5883638 | a2 | 100 | 1975.0394 |
| 270 | L6 | FAM1 VIC2 | 2.965509 | 1.542744 | a2 | 100 | 2038.2646 |
| 272 | L8 | FAM1 VIC2 | 2.4227138 | 3.102088 | Both | 100 | 1869.6477 |
| 274 | L10 | FAM1 VIC2 | 3.1533787 | 1.5406849 | a2 | 100 | 1925.7032 |
| 290 | M2 | FAM1 VIC2 | 0.4778691 | 1.4174258 | Undetermined |  | 2122.6763 |
| 292 | M4 | FAM1 VIC2 | 0.4941786 | 1.3713795 | Undetermined |  | 1863.7122 |

***** a1: Met/Met; a2: Val/Val; and Both: Val/Met.

**Table S2 Demographics and clinical characteristics of the sample cohort used for microarray experiments**

| **Variable** | **Total**  **(n= 26)** | **CAP**  **(n= 11)** | **Healthy**  **Control**  **(n= 15)** | **p-value** |
| --- | --- | --- | --- | --- |
| **Gender (n)**  Females  Males | 15 (58%)  11 (42%) | 7 (64%)  4 (36%) | 8 (53%)  7 (47%) | - |
| **Race (n)**  White | 26 (100%) | 11 (100%) | 15 (100%) | - |
| **Age**  Range (yrs) | 26.5 ± 7.01  (14 - 45) | 25.09 ± 5.07  (14 - 32) | 27.53 ± 8.17  (15 - 45) | 0.358 |
| **Body Mass Index (BMI)**  Range (kg/m^2^) | 25.15 ± 5.60  (18.82 - 43.22) | 24.57 ± 4.28  (20.19 - 35.07) | 25.57 ± 6.52  (18.82 - 43.22) | 0.643 |

Age and BMI values are reported as mean ± standard deviation.

**Table S3** Microarray differentially expressed genes for the BDNF group with fold change criteria (>2.0 and < -2.0) and unadjusted p-values (< 0.05) for a subset of 26 participants

| **Probeset ID** | **Gene Symbol** | **Gene Title** | **p-value (Val/Val vs. Met Carrier)** | **Fold-Change (Val/Val vs. Met Carrier)** |
| --- | --- | --- | --- | --- |
| 231979_at | --- | --- | 0.0212587 | 2.18091 |
| 219935_at | ADAMTS5 | ADAM metallopeptidase with thrombospondin type 1 motif, 5 | 0.00688026 | 2.01129 |
| 1554558_at | DCAF5 | DDB1 and CUL4 associated factor 5 | 0.00508387 | -2.01985 |
| 215574_at | --- | --- | 0.0375401 | -2.19516 |
| 234082_at | --- | --- | 0.0079731 | -2.41981 |
| 226303_at | PGM5 | phosphoglucomutase 5 | 0.046951 | -2.50208 |
| 231381_at | ESRG | embryonic stem cell related (non-protein coding) | 0.0258002 | -2.63939 |
| 231236_at | ZFP57 | zinc finger protein 57 homolog (mouse) | 0.0305216 | -2.7236 |
| 220784_s_at | UTS2 | urotensin 2 | 0.0127225 | -3.12385 |
| 213831_at | HLA-DQA1 /// LOC100507718 /// LOC100509457 | major histocompatibility complex, class II, DQ alpha 1 /// HLA class II histocompatibil | 0.0438425 | -10.6001 |
